# The social and economic burden on family caregivers for older adults in the Czech Republic

**DOI:** 10.1186/s12877-020-01571-2

**Published:** 2020-05-11

**Authors:** Petra Maresova, Sunwoo Lee, Oluwaseun Omolaja Fadeyi, Kamil Kuca

**Affiliations:** 1 Faculty of Informatics and Management, University of Hradec Kralove, Rokitanskeho 62, 50003 Hradec Kralove, Czech Republic; 2 Department of Geology, Faculty of Geography and Geoscience, University of Trier, Universitätsring 15, 54296 Trier, Germany

**Keywords:** Family caregivers, Home care, Disease, Seniors, Burden

## Abstract

**Background:**

In this paper, we analyse the economic burden of elder care in the Czech Republic by assessing how family members of senior citizens engage in caregiving. To do this, we examined the time and cost associated with caregiving as a function of age, gender, and income of the caregiver.

**Methods:**

A questionnaire comprising 17 items was used to gather data from 155 informal caregivers whose seniors are affiliated with 200 registered home care agencies. Spearman rank correlation was adopted to analyse how caregivers’ income, age and gender influences the costs accrued and time spent in caring for elders. The expenses are divided into direct and indirect. Publicly available sources are used to establish expenses on treatment and medication, which cannot be determined by the questionnaire survey.

**Results:**

Results show that around-the-clock care is the most common. Spouses of care recipients make up the highest number of caregivers, and women represent the largest portion of this group. Additionally, the more the time spent caring for an elder, the higher the cost associated with such care. The total annual burden of informal caregivers is determined by the sum of annual average indirect costs, amounting to € 36,888, and annual direct costs, amounting to € 2775, resulting in a total of € 39,663 per year.

**Conclusion:**

Czech social policymakers should begin to consider better packages for caregivers in the form of incentives and other monetary benefits to improve living standards and care for ageing seniors.

## Background

An increase in ageing populations often calls for reformulated policy resolutions that can cover social security and healthcare costs [1]. Healthcare and social systems in the Czech Republic are currently separated [2], which presents challenges in arriving at a systemic solution to the challenges associated with an aging [3].

Family members are most commonly those who take up the task of looking after the sick and the elderly [4]. This arrangement, however, takes its toll on the caregivers’ quality of life, as Metzeltin el al. (2017) [4] point out. Bevans et al. [5] add that the burden on family caregivers is multifaceted, including physical strain, emotional stress and financial difficulties attendant on providing long-term care for the chronically ill and/or handicapped. The overall wellbeing of the family caregiver is significantly influenced by the type of disease which the family member suffers from [6]. According to Kageyma et al. [6], the caregiver burden may be so heavy that the caregivers themselves may become chronically ill and eventually unable to continue providing care [7]. This particularly concerns spouse caregivers [8], who suffer from the prolonged stress of looking after their life partner who is losing self-sufficiency [9]. This already difficult situation is further aggravated when the patient suffers from early onset dementia, which is the most common diagnosis of older adults who are provided with informal care by their spouses. In these cases, the children of the couple also contribute to providing care for the ill parent [6].

Family caregivers in the labour force often fail to reconcile the demands of caregiving and a full-time job. This either causes them to reduce working hours or to leave their jobs in some cases. As such, there is little or no income at all for these caregivers. Spiess and Schneider [7] reported that female caregivers in the northern part of Europe reduced their working hours as soon as they started providing care or when their caregiving workload increased. Similarly, a number of studies have shown that one-third of caregivers found their regular employment compromised due to caregiving duties; some were ultimately forced to quit their jobs [8]. Trepel [9] noted that some working-age individuals decided not to take paid jobs, in order to devote themselves exclusively to caregiving. Others reduced their working hours to 20 h or fewer per week.

In the Czech Republic, more than 80% of seniors rely on care from their family members [10]. Therefore, there is an urgent need for a solution from policy makers, so that the burden of caregiving by family members can be reduced. In light of this prevailing socio-economic challenge, and given the lack of substantial literature related to caregiving for senior citizens by family members in Czechia, the current study seeks to provide an economic estimate of caregiving costs and time demands in the Czech Republic. Cost in this context is divided into to three main categories: direct medical costs, direct non-medical costs, and indirect costs.

Family caregivers make up more than 80% of long-term care providers in the Czech Republic [11], which is corroborated also by current studies abroad [12]. This means that the Czech Republic counts among European countries with the highest proportion of informal care provision; it is second only to Poland. When it comes to occasional care, it is provided from 97% by family members and friends, while daily care is provided from 78.5% by close family [13]. The primary caregivers are most often female. Research [14] indicates that in six European countries, 76% of family caregivers providing care for the elderly are women, aged 55 on average. Data for the Czech Republic show that women aged over 55, which is the largest group of informal caregivers [15], frequently drop their employment to be able to provide care for their family member. The employment rate in this group decreases to 65.3% for ages 55–59 and to 7% for ages 60 and above. In contrast, the employment rate of men of the same age groups does not undergo such dramatic change and amounts to 82% for ages 55–59 and 18.5% for ages 60 and above [16]. It is therefore women more often than men who become economically inactive for reasons of providing care to a family member and running a household; specifically, in the Czech Republic it is 319,000 women in contrast to 3200 men who leave their jobs for these reasons [17]. An entry-level caregiver job for caregivers with less than 3 years of experience is remunerated by an average salary of € 8228; whereas an experienced caregiver with eight and more years of experience receives an average salary of € 12,240 per year. These estimates were calculated based on data from an anonymous survey among employers and employees in the Czech Republic [18].

In comparison to super aged societies such as Japan, US, Germany, Czechia has a better situation, but the issue is also solved very intensively. Germany is currently one of five super-aged societies in the world, and its population of those age 65 and older will continue to grow. In order to address the growing need for care, the government has been working to broaden the scope and inclusivity of its LTC system, placing particular focus on improving conditions for both recipients and providers of home-based care.

This paper contributes in the area of knowledge, which type of care is currently used by seniors, to what extent and to which degree. Knowledge of these facts contributes to better plan future care.

## Methods

### Study design

The aim of this study is to determine the economic burden of informal caregivers. For the sake of comprehensiveness, the findings are complemented by data from the Institute of Health Information and Statistics (IHIS), the Czech Statistical Office (CSO) and the General Health Insurance Company (GHI). This allows to present a complete overview of the economic burden, including the expenses on medication and treatment (not only supplementary payments for medication as determined by the questionnaire survey).

*Questionnaire Survey.*

Data was collected using a questionnaire survey (see Appendix Table 10). The focus and structure of the questionnaire items were partly based on the study “Economic valuation and determinants of informal care to people with Alzheimer’s disease” [19], which was performed in Spain and aimed to determine the monetary value of the work delivered by informal caregivers for patients with Alzheimer’s disease. The study tested, for example, the following hypotheses: “the greater the degree of dependency, the more time invested in informal caregiving hours; the more formal services received at home, the less time invested in informal caregiving hours (substitutive services)”.

Based on this, the authors continued to test the relationships and dependencies that have been the subject of multiple previous studies, such as: Is there a dependency between gender and the time spent with the patient? Furthermore, the examined dependencies were focused in detail on the financial aspect in terms of expenses incurred by informal caregivers. The following questions were asked: Is there a dependency between the monthly expenses on formal care and the workload in hours of the informal caregiver? Is there a dependency between the time spent with the patient by the informal caregiver and the time spent with the patient by the formal caregiver? Is there a dependency between the time spent with the patient by the informal caregiver and the requirements of the patient on special equipment (chapter 3.3)?

The questionnaire comprised 17 items, 12 of which were closed-ended, requiring the respondent to select from a variety of options. The remaining questions were open in the sense of giving the option to provide “other reasons”, where the respondent could add free-form answers.

Questions included in the survey were selected in keeping with the methodology of [20] in three stages. The first stage consisted of generating questions and validating them in terms of content. The second stage was focused on constructing the scale. The scale was pre-tested in order to eliminate unnecessary items and to determine how many factors the scale is able to assess. Finally, the last stage tested the number of dimensions, reliability and validity.

Questionnaire items were divided into three sections. In the first section, respondents were asked to provide basic demographic information as well as information on their caregiving workload. The second section focused on care recipients’ details and costs incurred in treatment and care services, while the last section was concerned with the time and cost burden on caregivers. The use of a questionnaire ensured that the study was able to gather exact data directly from the care-givers themselves and relate that information to their overall well-being.

#### Publicly available sources

Publicly available sources were used to determine expenses on treatment and medication, which could not have been determined in the questionnaire survey. The reason is that the respondents are only aware of the amount of supplementary payments for medication which they are required to cover. This complementary data was available from the records of the General Health Insurance Company, which provided data in the following categories: outpatient, inpatient, transport, medication, medical aids and supplementary payments.

### Population, sample and sampling

The population for this study consisted of all 475 government-funded home care centres within the Czech Republic [21]. These centres have representatives who provide home care services to seniors who choose to remain with their families. Home care is a fundamental aspect of the Czech Republic’s long-term elderly care structure [22], mainly composed of some basic personal assistance services and a number of specialized services such as administering injections and drugs [10]. Care homes largely depend on government funding, except a few private homes funded by private organizations. Most seniors above 65 enjoy free insurance coverage, as is the case in many EU member states; hence, some or all services from care homes may be free of charge [10]. Care homes staff asked informal caregivers to fill in the questionnaire, so the respondents were informal caregivers.

Certain medical devices must be paid for or purchased by family members, but this is relatively rare.

Given our target population of 475 home care agencies, the selected sample size had to be a good representation of the population. This study therefore adopted the Creative Research Sytems‘sample size calculation technique [11], and using a confidence level of 95%, results showed that 200 was a sufficient sample to represent the entire population [12]. Since population density varies across the 14 regions of the Czech Republic [13], purposive sampling was done on the basis of population density, so that more care centres were selected from highly populated regions like Prague, Moravian-Silesian, and Central Bohemia than from low-populated regions like Plzeň, Vysočina, and South Bohemia. Table 1 shows the distribution of selected care centres across regions of the Czech Republic.

**Table 1:** Selected number of care homes per region

Region	Population density (per Km^2^)	Number of selected care centres
Liberec	135	16
Hradec Králové	115	14
Pardubice	112	14
Olomouc	123	15
Moravian-Silesian	227	20
South Moravian	159	18
Prague	2360	36
Central Bohemia	104	10
South Bohemia	62	5
Vysočina	75	5
Plzeň	73	5
Kalovy vary	92	7
Ústí nad Labem	154	18
Zlín	149	17
Total		200

Population density information has been derived from [14].

### Data collection

#### Questionnaire

A descriptive correlational research design [15] was adopted in order to check how caregiving time demands on family members relate to their finances and health. A total of 200 survey questionnaires were distributed to caregivers across the 200 selected care homes in regions of the Czech Republic. The study specifically considered caregivers providing home care to sick older adults (i.e., those who work in long-term care homes and also visit adults in their homes for the administration of drugs and injection). We did not consider homes where short-term care of seniors was carried out. Care homes were given the names HC-1, HC-2, etc., up to HC-200. We defined home care as the provision of nursing care, meals, and personal care, as well as administration of drugs and injections, among other activities.

Questionnaires were administered to the caregivers between December 2016 and July 2017 by a team of master’s diploma students led by Jakub Betka, a diploma student. The students had earlier been involved in a training course on data gathering, and retrieved data were duly scrutinized by the research team at the Faculty of Informatics and Management of the University of Hradec Kralove. Of the 200 questionnaires distributed, 180 were returned (yielding a 90% response rate). This figure was judged good based on Fincham’s findings [16]. Nevertheless, only 155 of the 180 questionnaires were correctly completed; hence, analysis was based only on correctly completed questionnaires. Data processing was subsequently carried out at the end of July 2017.

### Ethical notes

Permission was sought from the health care board in charge of regulating all long-term care homes within the Czech Republic. The authority to carry out questionnaire surveys across care homes was granted in May 2017, after a thorough check of the questionnaire’s content. As part of the questionnaire instructions, respondents were free to discontinue participation without prior notice, and each respondent had the choice whether or not to participate. Furthermore, an agreement was reached with care home authorities so that respondents’ identities were not divulged. The study was submitted to and approved by our institutional ethics committee, the Ethics Committee for Research at the University of Hradec Králové.

### Data analysis

Gathered data were first sorted into tables to determine the frequency of individual variables. For correlation between variables, the Spearman correlation coefficient was used, while the chi square test was adopted within the contingency tables. NCSS 2010 and IBM SPSS Statistics 20 software were both adopted for data analysis administration. Cost was grouped according to usage (see Table 2). Classification of the costs ensured that direct medical cost, for instance, was specifically used in the establishment and operation of the medical programme [17].

**Table 2:** List of types of costs for analysing socio-economic burden of caregivers

Types of costs		
Direct medical cost	Direct non-medical cost	Indirect cost
Administration of injection, outpatient, hospitalisation, transport and others medication, medical devices	Sum of monthly expenses for caregiver services (rehabilitation psychotherapy (psychologist), individual session, phone/email / skype session, massages, physical activity (walking, household chores etc.), mental activities, therapy courses, online skype therapy, home therapy, leisure activities, book readings, painting courses, patient education, complementary therapy, acupuncture, energetic therapy) calculated as average price per hour times number of hours	Derived from questionnaire responses (method of opportunity cost)
	Average monthly expenses for additional services (lending medical aids: adjustable bed wheelchair, walking frame, anti-decubitus mattresses, crutches, oxygenator, toilet chair) price per service	

It should be noted that only a few indirect costs were considered for the purpose of this study. For instance, costs related to loss of productivity of the patient or caregiver were not taken into consideration. The following expenditures were considered: medicines, treatments (inpatient and outpatient care, visits to a general practitioner), bedspace in a nursing home, caregiving services, and some miscellaneous services. An important aspect of our analysis was the calculation of informal care by caregivers using opportunity cost methods. Table 2 summarizes the different categories of cost and expenditure calculated under each of the cost groupings. The table clarifies the exact types of cost that family members have to bear so that the patient can receive adequate care.

#### Costs calculation

Total cost is calculated by the summation of direct medical, direct non-medical, and indirect costs.

- Direct medical costs; supplementary payments for medication + treatment + insurance companies costs
- Direct non-medical costs; caregiving services + additional services
- Indirect costs; caregiver time * caregiver wage

Indirect costs are calculated based on the questionnaire results using the opportunity cost method. Mathematically, indirect cost is given as:

- *Indirect cost* =*t*_*i*_ ∗ *w*_*i*_
- where ‘*w’* is the caregiver’s wage (see question no. 5 on the questionnaire), ‘t’ is the time spent by an elderly patient with a caregiver (see question no. 6 on the questionnaire), and ‘*i*’ is time period.

## Results

### Profile of caregivers

Table 3 provides an overview of study participants. Of the respondents, women accounted for 59% of all family care givers. The age of caregivers varied; more than half of caregivers were 51 years or older (57%), 6% were between 18 and 30 years (*N* = 10), 13% were between 31 and 40 years (*N* = 20), and 24% were between 41 and 50 years (*N* = 37). Nearly half of the respondents were partners/spouses of the patient (45.2%); 36.8% were children, 11% were grandchildren, and 7.1% were other relatives. More than 50% of the caregivers were employed (53.5%), 12.9% were unemployed, and 33.5% were retired. Unemployment is mainly the result of individuals having to quit their jobs to take up caregiving roles, as some 8.3% (13 out of 20) explained that they could not cope with combining the workload of caregiving and employment. It should be noted that there are provisions made by the government for unemployed caregivers, and they have access to other forms of finances, e.g., caregiver benefits, charity, and unemployment benefits, among others.

**Table 3:** Caregivers’ profile

	Respondents			
	Female		Male	
Age/Gender	**N**	**%**	**N**	**%**
18–30	3	3.3	7	7.7
31–40	17	18.7	3	3.3
41–50	22	24.2	15	16.5
51 +	49	53.8	39	42.9
Total	**91**		**64**	
Relationship with care recipients^a^	**N**		%	
Wife	38		45.2 (Spouse: husbands & wives)	
Husband	32			
Daughter	37		36.8 (Children: daughters & sons)	
Son	20			
Grandchild	17		11.0 (Grandchildren)	
Other relatives	10			
Professional caregiver	1		7.1 (Other relatives &)	
Caregivers’ social position^a^	**N**		%	
Employed	83		53.5	
Unemployed	20		12.9	
Retired	52		33.5	
Monthly income (EUR)^a^	**N**		%	
230–370	36		23.2	
370–450	39		25.1	
450–560	28		18.1	
560–740	36		23.2	
740 and more	16		10.3	

^a^share of the total number of respondents 155

56.7% of caregivers were 51 or older. At the time of data collection, approximately 54% of respondents were gainfully employed. Although wages did differ significantly, 25% of surveyed caregivers earned between €370–450 on a monthly basis. Only about 10% of caregivers earned an amount greater or equal to €740 monthly. These figures correspond to those of the Czech Statistical Office [18], where the average monthly retirement amount is slightly below €444.

Table 4 highlights the health challenges of the care recipient. Senile dementia is a common disease in older adults in the Czech Republic, with 16% of them requiring care (10.8% of all female seniors were diagnosed with senile dementia). Oncological diseases are also common in male seniors, at a rate of about 7%.

**Table 4:** Disease types of care recipients

Disease Type	Male		Female	
	N	%^a^	N	%^a^
Alzheimer’s disease	6	2.8%	11	5.2%
Asthma	–	–	2	0.9%
Bed-bound patients	–	–	1	0.5%
Blindness	–	–	2	0.9%
Cerebral palsy	2	0.9%	–	–
Coma	–	–	2	0.9%
Coronary artery disease	–	–	3	1.4%
Diabetes	12	5.6%	19	8.9%
Down syndrome	1	0.5%	–	–
Epilepsy	2	0.9%	2	0.9%
Haematopoiesis disease	–	–	1	0.5%
Heart disease	1	0.5%	3	1.4%
Hypertension	–	–	9	4.2%
Limb amputation	4	1.9%	3	1.4%
Limited mobility	7	3.3%	8	3.8%
Limited self-sufficiency	–	–	1	0.5%
Liver cirrhosis	–	–	2	0.9%
Lung disease	–	–	2	0.9%
Mental disease	1	0.5%	5	2.3%
Minor stroke	–	–	1	0.5%
Multiple sclerosis	2	0.9%	5	2.3%
Old age	14	6.6%	5	2.3%
Oncological diseases	15	7.0%	14	6.6%
Parkinson’s disease	1	0.5%	4	1.9%
Senile dementia	11	5.2%	23	10.8%
Severe multiple diseases	1	0.5%	–	–
Stroke	1	0.5%	1	0.5%
Tuberculosis	2	0.9%	1	0.5%

^a^expresses the share in the total number of respondents 155

Respondents listed more than one disease when answering questions related to diseases, mainly because the diseases listed in Table 4 tend to be related to other conditions. For instance, limb amputation can be a result of diabetes [23], and Alzheimer’s disease can be a result of old age [20], which is also connected to loss of self-sufficiency and mobility [24]. Overall, 47 other conditions were reportedly associated with each of the main diseases in Table 4.

### Time demands and costs of caregiving

#### Time demands

Table 5 provides information about the time demands reported by respondents. Time demands varied according to different caregivers’ job positions. Of the respondents, 43.5% reported providing constant care. Approximately 18% of respondents spent one to three hours per day on caregiving.

**Table 5:** Employment and caregiving time demands

	Time demands				
Caregiver and work position	1 - 3 h	1/2 - 1 h	4 - 6 h	6 - 8 h	Constant care (24 h)
Senior	2	1		2	9
Part time job	4		4	7	10
Did not affect employment	20	9	21	11	24
Left employment				1	17
Others	1	2		3	7
Total	27	12	25	24	67
%	17.5	7.8	16.2	15.4	43.5

In light of the above data, it is important to investigate how informal caregivers perform needed caregiving activities, regardless of whether their caregiving time demands interfere with their employment. In most cases, a long-term care agency representative works between one and 3 h daily to attend to care recipients’ technical needs, including administering medication, injections, and opiates (as per request of informal caregivers). Some caregivers use this break, when the patient is attended by a paid agency employee, to work part-time.

#### Home care service costs

**Fig. 1:**
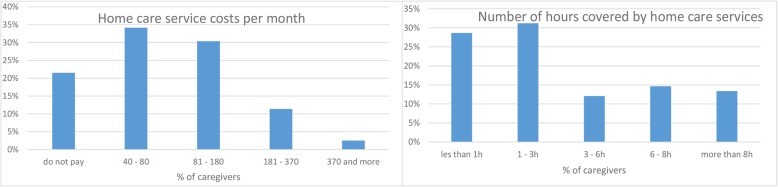
Home care service costs and caregiving time demands

Home care service from agency representatives comes at an additional cost for most respondents. The cost varies from as low as €40 to as much as €180 monthly. It is clear from Fig. 1 that while some people paid for those services, for 21% of all respondents the cost was borne by charity and often by the government. 31% professionals rendered home care services between one and 3 h daily; 29% individuals spent about 30 min to 1 h; 12% spent between three and 6 h; 15% six and 8 h; and 13% more than eight hours daily. The weighted arithmetic mean is €1852.

#### Medical device costs

Because of the wide range of health issues confronting care recipients, specific medical devices and services are often required to monitor and improve health conditions and quality of life. Table 6 details some of those costs, also showing the kind of payment accepted. Most services lasted between 50 and 90 min and were charged based on the number of minutes the care professional spent with the patient. For instance, a massage may cost as much as €37. Families of care recipients (79%) also needed to provide one-off payments for certain medical devices. Rehabilitative medical devices such as wheelchairs (43.8%), adjustable beds (45.8%), and walking frames (78.7%) were the most common. Complementary therapy and patient education (9.6%) cost the least of all medical devices/services.

**Table 6:** Pricing of healthcare services and products

Activity / Device / Service	Payment type	
	50–90 min	One-time payment
Rehabilitation	€ 29	–
Psychotherapy (psychologist)	€ 28	–
Individual session	€ 27	–
Phone / Email / Skype session	€ 30	–
Massages	€ 37	–
At home	€ 39	–
At studio	€ 35	–
Physical activity (walking, household chores etc.)	–	–
Mental activities	–	€ 53
Therapy courses	–	€ 59
Online Skype therapy	–	€ 48
Home therapy	–	€ 37
Leisure activities	–	–
Book readings	–	€ 6/month
Painting courses	–	€ 99/ course
Patient education	–	–
Complementary therapy	–	€ 39
Acupuncture	–	€ 42
Energetic therapy	–	€ 39
Yoga	€ 28	–
Adjustable bed	–	€ 33/month
Wheelchair	–	€ 17/month
Walking frame	–	€ 9/month
Other (option to add one’s own answer)	–	–
Anti-decubitus mattresses	–	€ 11 /month
Crutches	–	€ 4/ month
Oxygenator	–	€ 39/ month
Toilet chair	–	€ 8 / month

Source: consultations with organizations in respondents‘care homes

To determine the financial burden on caregivers, the costs of medical devices and services per month were calculated. 50% of the surveyed respondents spent between €20 and €50 monthly on medical devices and related activities; 28% claimed they spent between €50 and €70 monthly. A total of 22% households reported no costs for special equipment. In such cases, payment is often one-off, or devices were provided free of charge. In terms of the weighted arithmetic mean, it amounts to € 34 per month, which is € 412 per year.

#### Direct medical costs determined by the questionnaire results

This type of costs mostly comprises expenses on medication, hospitalisation, medical aids, etc. The questionnaire results were analysed to determine average monthly expenses of informal caregivers in terms of supplementary payments, including medication costs. The initial assessment of the questionnaire as to the clarity and comprehensibility of questions has indicated that it is appropriate to offer a range of possible supplementary payments. The results are provided in Table 7.

**Table 7:** Supplementary payments for medication

Supplementary payments for medication (€)		
Payment amount (€)	Number of respondents	Category average
20 or less	47	300
21–60	76	1000
61–100	21	2000
101 or more	11	3000

The weighted arithmetic mean across categories is € 6604. This amounts to an average of € 43 per one respondent per month, which is € 511 per year.

### Examining the relationship between variables

To arrive at robust results for the socio-economic burden of family caregivers in the Czech Republic, the following relationships were tested:

- Caregivers’ time demands and the financial burden for paid home-care services;
- Caregivers’ time demands and their monthly income; and
- Gender of caregivers and their caregiving time demands.

Correlation analysis shows that a moderate relationship exists between time demands on caregivers and the cost accrued in home-care services, i.e., *r* = 0.521 (*N* = 155), *p* < .01. This implies that higher caregiving time results in corresponding greater costs for home care services. Furthermore, it was observed there is no relationship between the demand on caregivers’ time and their incomes, i.e., *r* = 0.155 (*p*-value 0.054). This was also the case for the relationship between gender and time spent with elderly patients (*p*-value 0.768). A chi-square test was adopted to analyse the relationship between caregivers’ time demands and the need for special equipment. Result yielded a chi-test value of 18.587 at a probability value of 0.001.

### Cost analysis

#### Economic burden of informal caregivers

The economic burden from the perspective of informal caregivers comprises direct and indirect costs, which will be calculated based on the data from the questionnaire survey.

#### Direct costs

In terms of direct costs, the questionnaire survey results include the following items: home care service costs, medical device costs and supplementary payments for medication. The average annual costs of medication amount to € 511 per year, for medical devices it is € 412 and for formal care it is € 1852 in the weighted arithmetic mean per year. The total amounts to € 2775 per year.

#### Indirect costs

Based on the questionnaire data, the following results were calculated:

- Average time “t” of all employed/unemployed = 9.85 h/day,
- Average time “t” of all respondents (including seniors) = 2.78 h/day.

In the same vein, average caregivers’ wages on the time spent with seniors. Finally, indirect cost was calculated by multiplying time and wage according to the opportunity cost method. Total indirect costs determined by questionnaire results are as follows:

- € 2806 / month excluding retired caregivers,
- € 85,576 / month including retired caregivers.

For both categories, the average amount calculated equals €3074 /monthly. General indirect costs based on publicly available sources were also calculated using the average hourly rate of caregivers (€4.7/h). According to public records, 70% of elderly patients receive care at least 10 h per week, while others receive more (20 h weekly). As such, the annual cost of care is thus estimated at €3375 per month.

The total annual burden given by the sum of both expense groups is therefore € 36,888 (annual average indirect costs) and € 2775 (annual direct costs), which amounts to a total of € 39,663 per year.

### Costs of treatment and care – societal perspective

In order to assess the overall burden, not only as seen from the perspective of informal caregivers, the data from the questionnaire survey are complemented by data from publicly available sources, so as to complete total direct costs, which means not only supplementary payments for medication but the total costs of medication, outpatient treatment, etc. Data from health insurance companies have been included as the companies were able to provide exact costs of medications, information otherwise difficult to come by. The authors are aware that at this moment, a certain bias occurs because with respect to the protection of personal data, it is not possible to connect the expenses of a specific patient with the data provided by the health insurance company. Therefore, the parameter of the average cost per person with a specific diagnosis was selected, using the diagnoses most often mentioned in the questionnaire survey.

Average costs of healthcare per one person in 2016 were CZK 25,944 per year (GHI – Yearbook, 2016). This number includes outpatient care, hospitalisation, transport, medication and medical aids. To complement the data on direct costs, the following most common diagnoses were selected: senile dementia and Alzheimer’s disease (nervous system diseases), diabetes (endocrine and metabolic diseases) and cancerous diseases (neoplasms). See Table 8 for an overview.

**Table 8:** Average direct costs of selected diseases according to GHI

Costs as of 2016	Senile dementia, Alzheimer’s disease	Diabetes; Neoplasms	Cancer; and Nervous system diseases
Average costs of health insurance companies per person	€ 424	€ 221	€ 628

Table 9 indicates the relationship of data from the questionnaire survey and data obtained from publicly available sources.

**Table 9:** Calculation of total costs

Questionnaire results		Public records	
Direct costs			
Medical costs			
Cost type	€ / year	General Health Insurance Company – Yearbook 2016	€ / year
Supplementary payments: medication and treatment	511	I. Diabetes; Neoplasms	221
		II. Cancer; and Nervous system diseases	628
		III. – Senile dementia, Alzheimer’s disease	424
		Average^a^	422
		Total average^b^	1015
Non-medical costs			
Caregiving services	1852		
Medical device and equipement	412		
Total	2264		
Indirect costs			
Including retired caregivers	40,113	Total general average^c^	3375
Excluding retired caregivers	33,680		
Total on average	36,888		

^a^ Average direct costs of the three most popular segments selected from the questionnaire;

^b^ Average direct costs for one insured patient (GHIC, 2016);

^c^General calculation using the opportunity cost method and public records

I, II, III – the most popular categories from questionnaire.

The total annual burden from societal perspective is given by a sum of both groups (direct and indirect costs), or more specifically, by a sum of direct medical costs (€ 1,015), direct non-medical costs (€ 2264) and indirect costs (€ 36,888), which amounts to *€*40,167/*year*.

## Discussion

In studying the burden on family caregivers in the Czech Republic, it has been found that informal caregivers are mostly represented by daughters and wives. These results correspond to the situation abroad, where, for example, XX [25] points out that most caregivers are women (87%), which includes daughters, daughters-in-law and wives. The need for care varies among individuals. While some only need help with paperwork and purchasing groceries [21], others require all-around care all day, every day. In our study, approximately 44% of caregivers provided around-the-clock care. Disease diagnosis largely determines how long a caregiver will attend to a sick senior. For instance, dementia patients who also have severe impairment require more than twice the amount of care time as patients with minor stroke and other milder diseases [26]. The OECD [21] explained that time demands of carers vary from one country to another; this study shows that caregivers in Czech Republic are like those in Korea, who also spend 20 or more hours per day caring for sickly seniors. Our study findings corroborate Czech statistics [27] showing that many caregivers are family members; within this group, 58.9% are women and represent the highest number of family care givers. The current study further supports the OECD report from 2011 on the participation on women in informal care. The results are also consistent with that of Rezende et al. [28], who noted that family caregivers are mostly middle-aged and elderly women, either daughters or wives of the care recipient. Middle-aged male carers are few; nevertheless, there are more male caregivers aged 75 and above than females [21].

According to Hiseman and Fackrell [29], care recipients generally prefer family members to rotating hospital or hospice staff. In terms of total indirect and direct costs, which represent the overall monetary burden borne by caregivers, caregivers in the Czech Republic incur a little more than €40,000 annually, and women likely bear more of the burden in terms of both monetary and psychological cost [30–34].

The research has shown that the total annual burden of informal caregivers is given by a sum of both cost groups, € 36,888 (annual average indirect costs) and € 2775 (annual direct costs), which amounts to a total of € 39,663 per year. Direct costs include supplementary payments for medication, which correspond to € 511 per person per year; furthermore, home care service costs, which correspond to € 1852 per year; and medical device and equipment costs, which correspond to € 412 per year. We observed that 50% of respondents spent between €20 and €50 monthly on medical devices or related services. This finding is in line with the work of Mathieu-Fritz and Guillot [27], who noted that medical practitioners often expect their patients to be in possession of devices that will help manage underlying conditions after initial treatments. An additionally 28% of all family caregivers pay between €55 and €95 monthly for such services. Larissa Schwarkopf (2011) [35] and her team conducted a research with 383 selected patients showing signs of dementia. The specification of costs was based on a division into informal and formal care. Informal care costs were represented by the average time spent by the caregiver with the patient per day. Total costs were made up from 80.2% by informal care, which includes loss of profit or opportunity costs. The remaining mere 19.8% was made up by costs on formal health care. Average annual costs of mild dementia were found to be 39,986 €; while with moderate dementia, the costs were 62,831 €. It is of interest that with patients suffering from moderate dementia, most expenses are incurred on care provision, while in terms of medical care, the costs remain about the same. Gerves et al. (2014) [36] focused in their research on Alzheimer’s disease patients aged 60 and above. Two methods were applied to determine indirect costs: the proxy good method and the method of opportunity costs. The proxy good method is based on accounting for the time spent on informal care provided by the caregiver to the patient. This time is expressed in terms of the market price of an approximate substitute with respect to the tasks performed. On the other hand, the method of opportunity costs is based on the loss of profit derived from activities which the caregiver used to do before becoming a care provider. The average monthly costs estimated with the proxy good method range between 1454 € and 3373 €, depending on the severity of dementia, the average annual costs then amount to a range of 17,448 € to 40,476 €. The method of opportunity costs delivered higher average monthly results, which equalled to 1823 € for patients with mild dementia, corresponding to 21,876 € per year. With more severe stages of dementia, the average monthly costs were estimated as 4288 €, corresponding to 51,456 € per year. Costs of informal care make up approximately 70–76% of the total costs. Overall, the costs calculated in the present study correspond to the findings of the above-cited studies, even though our study does not focus on one disease only. The reason is the generally limited ADL of patients across diseases.

Furthermore, it should be pointed out that in calculating the costs, one of the items was completed by data from publicly available sources. The data in question was obtained from the General Health Insurance Company, which expanded the direct medical costs by adding costs on treatment and unreduced costs on medication. The difference between the unreduced costs on medication and the supplementary payments for medication as reported by the questionnaire respondents amounts to € 1015. The total annual burden from societal perspective is given by a sum of both groups (direct and indirect costs), or more specifically, by a sum of direct medical costs (€ 1015), direct non-medical costs (€ 2264) and indirect costs (€ 36,888), which amounts to € 40,167/year.

The decision to include further direct costs and the value of the result must be interpreted very carefully. This calculation is limited particularly by the fact that the direct costs are not linked to specific individuals in the questionnaire survey, rather, it represents average medical costs per person per year in the Czech Republic. The calculation therefore serves to complement the results obtained from the questionnaire. Considering the fact that direct medical costs represent only a small part of the total annual burden, we do not consider the possible bias to be large enough to disqualify the results. Another limitation of this study is the fact that it does not specify costs with respect to different diseases. One of the reasons is that the patients attended by informal caregivers suffer from multiple diseases, and it would be difficult to distinguish different needs for different diseases. Another reason is that a significantly larger respondent sample would be required to research combinations of diseases. However, given the aim of this study, a sufficiently large sample was obtained, especially with respect to ADL.

## Conclusion

Quality of life for Czech caregivers (including work life, financial situation, and health) is often at stake, particularly for those who engage in 24-h care. Although a number of people claim that caregiving does not affect their work life, many of them rarely engage in intensive caregiving, as explained by the OECD [21]. The excessive work burden and continuous financial indebtedness may lead to eventual financial breakdown for most caregivers in the Czech Republic. If this occurs, women will likely be the most affected. Caregiving can be stressful, especially when the care recipient is incapacitated and can barely take care of his or her own daily needs. This affects the caregiver’s mental well-being and socioeconomic status.

In the Czech Republic, senior day care centres, or long-term care centres, meet the needs of care recipients in all aspects, physically, socially and psychologically. These centres are able to provide daily routine tasks such as prescribing medication, preparing meals, and assisting with hygiene. Patients also have the opportunity to develop and maintain their social interactions with other patients.

This study highlights the disadvantages of family caregiving within the Czech Republic. While the country continues to strive to effectively care for older adults with long-term illness as well as for their caregivers, a centralized coordination of these efforts is still lacking at the national level.

The study also provides information on the use of healthcare, formal and informal care. This information can contribute to the planning of selected types of care, not only in the Czech Republic, but also in other economies affected by the aging population.

This study has some limitations. First, stages of diseases were not factored into the cost of treatment, which impacts the accuracy of the calculations of cost and burden on caregivers. Secondly, data from a number of public databases were used, and these may include estimated, not exact, figures, raising doubts about the accuracy of interpretations. Other financial burdens, such as the cost of food items and other household needs, were not considered. Despite its limitations, results of this study show the importance and burden of informal care.

The implications of this study for improved medical education, especially related to the health and well-being of family caregivers, are clear. Policy makers must develop ideas to help maintain the health of family caregivers. The government and policy makers should experiment in linking health care and social systems within Czechia, which might prove to be a long-lasting solution to family caregiving in particular and caregiving a whole.

## Supplementary information

**Additional file 1: **Appendix **Table 10.** Survey question.

## Data Availability

The datasets used and/or analysed during the current study are available from the corresponding author on reasonable request.
