# Supplementary material for: The social and economic burden on family caregivers for older adults in the Czech Republic
Source: BMC Geriatr. 2020 May 11;20:171. doi: 10.1186/s12877-020-01571-2 (PMC7216393; doi:10.1186/s12877-020-01571-2)
Supplement: Supplementary file 1 — Additional file 1: Appendix Table 10. Survey question. [file 12877_2020_1571_MOESM1_ESM.docx]

Appendix

**Table 10 Surevey question**

| **Part 1. General information** | |
| --- | --- |
| Gender | - Male - Female |
| A person's relationship to a patient | - Husband/Wife - Son/Daughter - Grandchildren - Other __________ |
| Age | - 18-30 - 31-40 - 41-50 - 51 and more |
| Your current job status | - Full time job - Jobless - Senior - Other |
| What is your monthly income (CZK) | - 6 000 – 8 000 - 8 000 – 10 000 - 10 000 – 12 000 - 12 000 – 15 000 - 15 000 – 20 000 - 20 000 and more CZK |
| How many hours a day do you spend with the patient? | - ½-1 h - 1-3 h - 4-6 h - 6-8 h - Continuous care (24 hours) |
| Did you have to reduce your workload due to the patient? | - I partially gave up my job - I completely gave up my job - It didn't limit me in my work process - Other (more comments) |
| Did you have to reduce your workload due to the patient? | - I partially gave up my job - I completely gave up my job - It didn't limit me in my work process - Other (more comments) |
| **Part 2. Diagnosis, Nursing services and pharmacy costs** | |
| What patient is suffering from the disease? |  |
| The patient needs special equipment | - Yes - No |
| Do you regularly pay for a care service? | - Yes - No |
| How many hours per day does the caregiver spend with the patient? | - ½-1 h - 1-3 h - 4-6 h - 6 and more |
| How much a month do you pay for care services (CZK)? | - 1 000-2 000 - 2 000-4 000 - 5 000-10 000 - 10 000 and more |
| Does the patient regularly take some kind of medication? | - Yes - No |
| Does the insurance / hospital pay you medication? | - Yes - No - Partially |
| How much additional are you paying monthly for drug payments, or drugs (CZK)? | - To 500 - 500–1500 - 1500–2500 - 2500 – and more |
| \| Would any of the activities and aids listed below help improve or improve the current state of the disease? \| It would be appropriate but not guaranteed \| Is ensured \| \| --- \| --- \| --- \| \| Rehabilitation \|  \|  \| \| Psychological treatment \|  \|  \| \| Massage \|  \|  \| \| Physical activity (walking, housework, etc.) \|  \|  \| \| Psychic Activities (Meditation) \|  \|  \| \| Fun Activities (Reading Books, Painting, Musical Instrument) \|  \|  \| \| Patient Education (Disease) \|  \|  \| \| Complementary therapy \|  \|  \| \| Adjustable be \|  \|  \| \| Pushcart \|  \|  \| \| Walker \|  \|  \| \| Other (write)…………………………………… \| \| \| | |
| How much does the above-mentioned additional services cost you per month (CZK)? | - 500 – 1 000 - 1 000 – 1 500 - 1 500 -2 500 - 2 500 – and more |
